# Improved Selectivity in 7 T Digit Mapping Using VASO-CBV

**DOI:** 10.1007/s10548-022-00932-x

**Published:** 2022-12-14

**Authors:** Ícaro A. F. de Oliveira, Jeroen C. W. Siero, Serge O. Dumoulin, Wietske van der Zwaag

**Affiliations:** 1 Spinoza Centre for Neuroimaging, Meibergdreef 75, 1105 BK Amsterdam, The Netherlands; 2 Experimental and Applied Psychology, VU University, Amsterdam, The Netherlands; 3 Computational Cognitive Neuroscience and Neuroimaging, Netherlands Institute for Neuroscience, Amsterdam, The Netherlands; 4 Experimental Psychology, Helmholtz Institute, Utrecht University, Utrecht, The Netherlands; 5 Radiology, Utrecht Center for Image Sciences, University Medical Center Utrecht, Utrecht, The Netherlands

**Keywords:** Vascular space occupancy, VASO, BOLD, Digit, Selectivity, Somatotopic, 7 T, High-resolution, Specificity

## Abstract

Functional magnetic resonance imaging (fMRI) at Ultra-high field (UHF, ≥ 7 T) benefits from significant gains in the BOLD contrast-to-noise ratio (CNR) and temporal signal-to-noise ratio (tSNR) compared to conventional field strengths (3 T). Although these improvements enabled researchers to study the human brain to unprecedented spatial resolution, the blood pooling effect reduces the spatial specificity of the widely-used gradient-echo BOLD acquisitions. In this context, vascular space occupancy (VASO-CBV) imaging may be advantageous since it is proposed to have a higher spatial specificity than BOLD. We hypothesized that the assumed higher specificity of VASO-CBV imaging would translate to reduced overlap in fine-scale digit representation maps compared to BOLD-based digit maps. We used sub-millimeter resolution VASO fMRI at 7 T to map VASO-CBV and BOLD responses simultaneously in the motor and somatosensory cortices during individual finger movement tasks. We assessed the cortical overlap in different ways, first by calculating similarity coefficient metrics (DICE and Jaccard) and second by calculating selectivity measures. In addition, we demonstrate a consistent topographical organization of the targeted digit representations (thumb-index-little finger) in the motor areas. We show that the VASO-CBV responses yielded less overlap between the digit clusters than BOLD, and other selectivity measures were higher for VASO-CBV too. In summary, these results were consistent across metrics and participants, confirming the higher spatial specificity of VASO-CBV compared to BOLD.

**Supplementary Information:**

The online version contains supplementary material available at 10.1007/s10548-022-00932-x.

## Introduction

Functional MRI (fMRI), based on the blood oxygenation level-dependent (BOLD) effect, is the most popular tool for mapping brain activity (Kim and Ogawa 2012). Recent advanced scanner technology, including ultra-high field (UHF) magnetic field strengths, improved image acquisition techniques and advanced analysis tools have enabled researchers to investigate the human brain at a sub-millimeter scales (Dumoulin et al. 2017; Norris and Polimeni 2019; Uğurbil 2021; Uludağ and Blinder 2016). In UHF-fMRI, there are two crucial gains for fMRI, the increased temporal SNR and the functional BOLD contrast that increases supra-linearly with field strength (Cai et al. 2021; van der Zwaag et al. 2009). Because of its high sensitivity to deoxyhemoglobin variations and widespread availability, gradient-echo (GRE) BOLD remains the most widely used contrast in fMRI. Unfortunately, the GRE BOLD signal is predominantly driven by the (large) draining vessels, resulting in a biased measurement towards the superficial cortical layers (Markuerkiaga et al. 2016; Uludaǧ et al. 2009; Yacoub and Wald 2018). Nevertheless, different MRI contrasts are sensitive to other physiological variables that can be used to study brain function, like cerebral blood flow (CBF) with Arterial Spin Labeling (ASL) (Kashyap et al. 2021) or cerebral blood volume (CBV) with Vascular Space Occupancy (VASO) techniques (Huber et al. 2017).

The human brain contains multiple homunculi, or orderly body representations, in the sensory and motor cortices (Penfield and Boldrey 1937). The individual body parts within these somatotopic maps occupy small parts of the cortex, integrating signal from a variable number of neurons (Gardner and Costanzo 1980). Visualizing somatotopic maps in humans with fMRI has benefited from the advent of UHF fMRI. The higher SNR at UHF can be used to acquire images with higher spatial and temporal resolution. Higher spatial resolution (smaller voxel sizes) reduces partial volume effects (PVE), enabling researchers to more accurately map the human somatotopic organization. Recent studies demonstrated individual finger movements in the primary motor (M1) and primary somatosensory (S1) areas, notably at the individual level (Besle et al. 2014; Kolasinski et al. 2016; Sanchez-Panchuelo et al. 2010; Schellekens et al. 2018; Stringer et al. 2011). In addition, some studies have focused on showing that the spatial pattern of BOLD activation at 7 T reflects the patterns of underlying neural activity (Martuzzi et al. 2014), with a direct comparison with electrocorticography (ECoG) (Siero et al. 2014). More recently, Huber and colleagues used the higher specificity of VASO-CBV to distinguish two mirrored topographical representations of digits in the primary motor cortex (Huber et al. 2020).

These studies show that high-resolution fMRI is a useful tool to study the somatotopic organization. The investigation of pathological conditions such as movement disorders or any disturbed sensory representation in neurological disorders like dystonia (Butterworth et al. 2003) also benefits from these methodological advances to map and quantify the organization of motor function (Marquis et al. 2017). Moreover, a qualitative and quantitative assessment of the changes in the cortical organization induced by these pathological conditions and the brain plasticity is essential to understanding the underlying mechanisms of brain disease. It may be relevant for developing or evaluating novel therapeutics or intervention strategies (Olman et al. 2012; Serino et al. 2017).

Because individual digit movements selectively activate distinct patches of the cortex, it is possible to quantify the degree of selectivity to one digit movement and the relationship of the cortical region to the movement of other digits (Akselrod et al. 2017; Martuzzi et al. 2014; Olman et al. 2012). This measurement is called selectivity (or response selectivity), and it can therefore be used to quantify the cortical overlap between adjacent digit clusters. There are, however, different contributions to the overlap, which can be separated in ‘neural’, ‘vascular’, and ‘methodology' related overlap. For example, the methodological aspects, such as the partial volume effect (PVE), head motion, and smoothing, are image acquisition-related and contribute to the measured overlap. The vascular overlap contribution arises from the blood pooling effect, which limits the spatial specificity of the BOLD response (Menon 2002; Turner 2002). The neuronal overlap contribution arises from the notion that a specific neuronal population distributed in a part of the motor cortex, for example, does not encode a single finger's movement. Instead, they may contribute to the movement of several fingers (Gardner and Costanzo 1980; Georgopoulos et al. 1999).

The Vascular Space Occupancy (VASO) method is advantageous since it promises higher specificity than BOLD due to reduced draining vein contamination (Huber et al. 2014; Jin and Kim 2008; Lu et al. 2013), especially for high spatial resolutions (Huber et al. 2020, 2017). In this context, ‘specificity’ indicates that the VASO technique is less sensitive to signals arising from large veins. The fine-scaled digit representation maps in the somatosensory cortex will likely benefit significantly from more spatially specific measurements. Here, we simultaneously measured VASO-CBV and BOLD responses at sub-millimeter resolution using a double 3D-EPI VASO sequence (Huber et al. 2014; Oliveira et al. 2021) during the execution of individual finger movements using block-designed stimuli. We hypothesize that the higher specificity of VASO-CBV images will result in higher response selectivity due to reduced vascular signal contributions. Our approach is notably different from recent studies that show the higher spatial specificity of VASO-CBV across cortical layers (Huber et al. 2017) or cortical columns (Huber et al. 2020). Here, we test that selectivity with several voxel-based measures.

## Materials and Methods

### Participants

Six healthy individuals (4 females mean age 30 ± 4 years) with no history of neurologic or orthopedic conditions participated in the study. All participants provided written informed consent before participating after being informed of the experimental procedures, and the local Medical Ethical Committee approved the study.

### Stimulus

The functional study was conducted in a single session. The participants performed the same task three times, once for each digit (thumb (D1), index (D2), and little (D5)) of the right hand. The task consisted of flexing one finger from an extended position periodically following a visual cue. The visual cue was projected on a screen at the end of the scanner’s bore, which the participants viewed using a mirror. The visual cue was generated in ‘Matlab’ (The MathWorks, Natick, United States) using the ‘Psychophysics Toolbox Version 3’ (Brainard 1997; Kleiner et al. 2007). The task paradigm consisted of 30 s of baseline with one-minute blocks: 30 s paced flexing-extension at 1 Hz and 30 s rest, repeated ten times, resulting in 10 min and 30 s of acquisition for each digit (defined as one run). For this task, flexion of D5 usually resulted in co-movement of D4 and the distal phalanx of D3. Nevertheless, we refer to this movement as ‘D5’. All participants underwent approximately 32 min of functional data recording, encompassing three 10-min runs, one for each digit. A functional localizer was used prior to the three-digit acquisition runs to ensure the left primary sensorimotor areas coverage. The stimulus paradigm consisted of fingertapping with 30 s of baseline, then alternating between 12 s of movement, and 24 s of rest, using a 3D EPI gradient echo sequence.

### MR Sequences

The Slice Selective Slab Inversion VASO with 3D EPI readout (Huber et al. 2014; Oliveira et al. 2021) was implemented on a 7 T MRI scanner (Philips Healthcare, Best, The Netherlands) using an eight-channel transmit coil and a 32 channel receive coil (Nova Medical Inc, Wilmington, United States) with B1 shim settings from a previous study (Oliveira et al. 2021). In addition, an adiabatic inversion TR-FOCI pulse was used to ensure an effective inversion with reduced B1 + inhomogeneity (Hurley et al. 2010). The timing parameters for the interleaved acquisition were TI_1_/TI_2_/TE/TR = 1100/2340/24/3000 ms. Data were acquired with a nominal in-plane resolution of 0.79 mm and nominal slice thickness of 1.5 mm (0.79 × 0.79 × 1.5 mm^3^), FOV = 140 × 140 × 20 mm^3^, matrix size = 176 × 176, 13 slices, partial Fourier factor = 0.78 in the phase encoding direction, BW_readout_ = 87 Hz, and SENSE_inplane_ factor = 2.7.

### Data Analysis

The preprocessing steps consisted of a separate motion correction for BOLD and VASO-CBV data using SPM (Statistical Parametric Mapping), followed by the BOLD correction to minimize the extravascular BOLD signal present in the VASO images (Huber et al. 2014). We used the NORDIC PCA denoising technique (Moeller et al. 2021; Vizioli et al. 2021) before the head motion correction step to increase the tSNR and accuracy of the functional maps. We first saved the magnitude and phase data from VASO-CBV and BOLD. Both data were denoised separately using version 1.1 (v.1.1) of the publicly available implementation (https://github.com/SteenMoeller/NORDIC_Raw). No separate noise scan was acquired. No additional smoothing or temporal filtering was applied to minimize the loss in specificity. The alignment across the three-digit runs was conducted as part of the motion correction. After that, we computed z-score maps for each finger movement using FEAT in FSL (v.6.0). Statistical differences between VASO-CBV and BOLD were assessed using Paired t-tests in R (R Core Team 2020).

The region of interest (ROI) definition was based on VASO-CBV and BOLD activated voxels as described below. M1 and S1 masks were drawn manually based on anatomical landmarks, i.e., the pre and post-central gyri. We included all voxels in the M1 and S1 regions that responded to the stimulation with a z-score greater than 2.5 for at least one digit in VASO-CBV and BOLD data. We did not attempt to focus on any specific Brodmann areas.

We calculated the Dice Similarity Coefficient (DSC) and Jaccard Similarity Coefficient (JSC) for each digit cluster-pair (D1–D2, D1–D5, and D2–D5). Both metrics are overlap-based and often used to validate segmentation boundaries (Carass et al. 2020), and here were used to quantify the overlap between digit clusters. We defined these clusters from the statistical z-score maps for the movement of each digit within the M1 and S1 ROI. Next, a ‘winner-takes-all’ approach was used to divide the ROIs into digit clusters to obtain binary (non-overlapping) digit representation maps and calculate the average z-scores of the dominant digit and the non-dominant digits (e.g., response to movement of D2 in the D1 ROI).

Finally, we used two distinct approaches to quantify the selectivity: (1) a general approach called ‘overall selectivity’ (OS). For the *OS* approach, we divide the maximum z-score between all three digits by the sum of the z-scores to all three digit movements. The advantage of the OS measure is the straightforward calculation because it does not require a winner-takes-all step (Eq. 1). However, the disadvantage is that negative z-scores in the non-dominant digits (a common occurrence in somatotopic maps) can lead to a division by 0 and excessive selectivity values (see Supplementary Fig. 1). Approach (2) is a more controlled measure, called ‘digit selectivity’ (DS). Voxels were assigned to a specific digit for the DS approach using a winner-takes-all approach, followed by Eq. (2) to quantify the selectivity per digit. In (2), we take the mean difference between the response of the thumb (D1), index finger (D2), and the little finger (D5) divided by the response of the thumb (D1). The benefit of the *DS* measure is that it avoids the division by zero or by negative values (see Supplementary Fig. 1). Supplementary Fig. 2 provides a graphical explanation of the selectivity metrics used in the present study.

1 $$Overall\, Selectivity = \frac{{\max \left( {D_{1} ,D_{2} ,D_{5} } \right)}}{{\sum {\left( {D_{1} ,D_{2} ,D_{5} } \right)} }}$$

2 $$Digit \,Selectivity \left( {D1} \right) = \frac{{0.5 \cdot \left( {\left( {D_{1} - D_{2} } \right) + \left( {D_{1} - D_{5} } \right)} \right)}}{{D_{1} }}$$

## Results

**Fig. 1:**
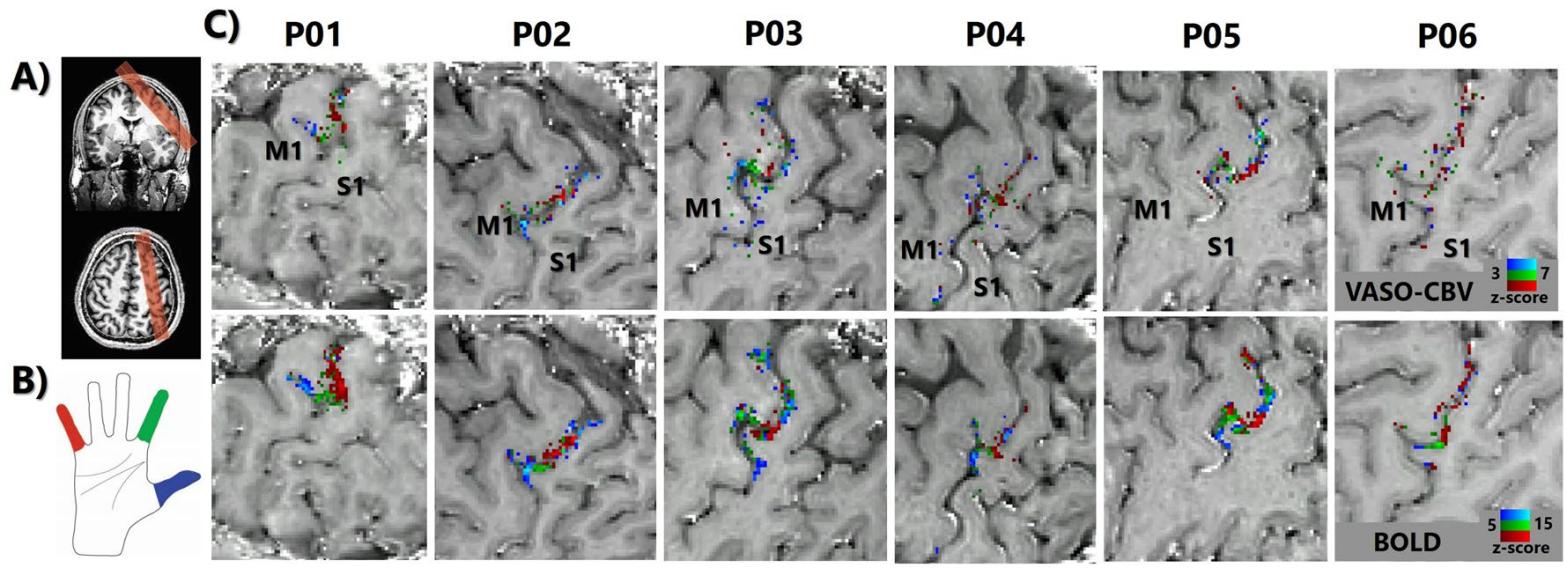
Overview of the acquisition method and the topographic digit mapping of a subset of fingers (D1, D2, and D5) using VASO-CBV and BOLD in the 6 participants. **A** We carefully positioned the imaging slab (0.79 × 0.79 × 1.5 mm^3^) to cover the left primary sensorimotor area. **B** The participants performed individual finger movement on a block-designed task, using thumb (blue, D1), index (green, D2), and little (red, D5). **C** Each voxel was assigned to a single digit using a winner-take-all algorithm, creating a subset of digit representation maps. The digit representations are orderly organized (thumb-index-little) along the central sulcus, predominantly in S1. No smoothing has been applied on VASO-CBV or BOLD data

Robust VASO-CBV and BOLD responses in M1 and S1 were detected in all six participants (Fig. 1). Figure 1 shows example slices of digit representation maps for all six participants. The maps show distinct activation patterns for movement of each of the three digits for this block-design task, organized in an orderly fashion (thumb-index-little finger) along the central sulcus and predominantly in S1. A secondary representation of the index and thumb, superior to the little digit representations, can be seen for subjects P02-P05. The VASO-CBV responses are smaller in amplitude and cluster size than the BOLD responses. In Fig. 1, the threshold (Z-score_VASO-CBV_ from 3 to 7 and from Z-score_BOLD_ from 5 to 15) is adapted to allow a visual comparison of the activation patterns.

**Fig. 2:**
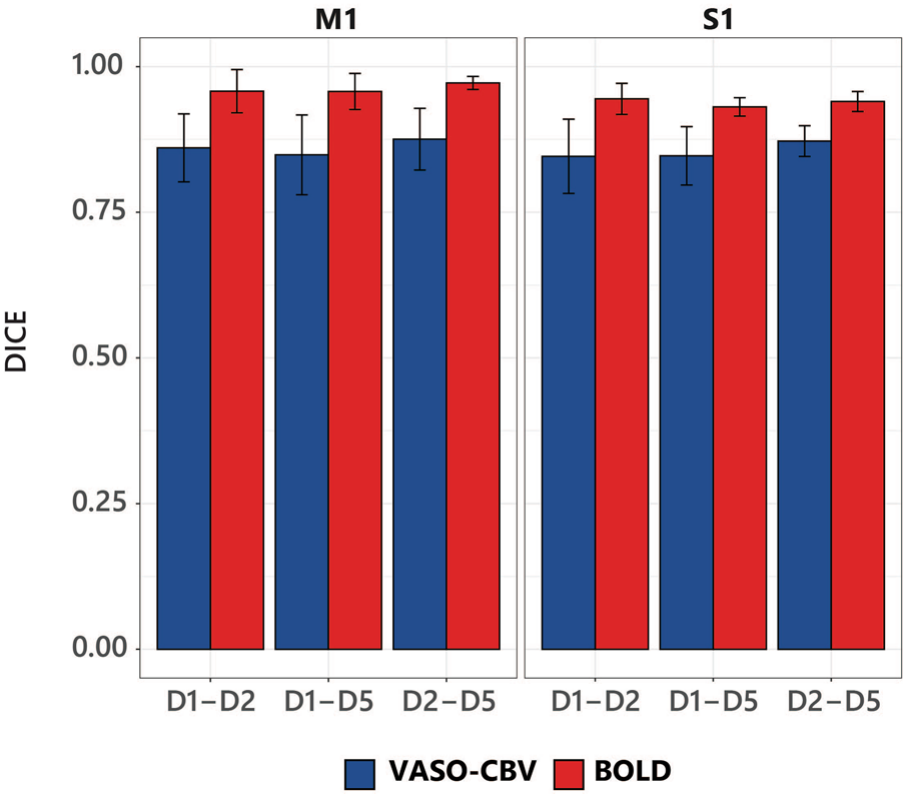
DICE similarity coefficients (DSC) for each pair-digit cluster in S1 and M1. BOLD yielded higher scores than VASO-CBV (Paired t-test, p < 0.05) in both regions. A higher similarity score represents a higher overlap between each digit pair. The error bar is the standard deviation. Please see Supplementary Fig. 3 for Jaccard measurements

The quantification of the spatial similarity of each digit-pair is depicted in Fig. 2. The DSC of each pair of digit clusters represents the overlap between that pair of response patterns. The pair-digits behave similarly for the M1 and S1 regions, with BOLD yielding consistently higher DSC values and, therefore, higher overlap than VASO-CBV. There was a significant difference between VASO-CBV and BOLD for both M1 and S1 regions for all three digit pairs (Paired t-tested, Bonferroni corrected). For S1, p-adjusted = 0.0066 for (D1–D2), p-adjusted = 0.015 for (D1–D5) and p-adjusted = 0.0053 for (D2–D5). For M1, p-adjusted = 0.00099 for (D1–D2), p-adjusted = 0.0049 for (D1–D5), p-adjusted = 0.019 for (D2–D5). The DSC scores were similar between digit-pairs for both ROIs, with a significant difference for the DSC score in the D2–D5 for BOLD (Paired t-test, p < 0.05). Additionally, we used a Jaccard Similarity Coefficient (Supplementary Fig. 3) on the same cluster in the same manner, and the results were nearly identical, with BOLD yielding a significantly higher overlap than VASO-CBV (Paired t-test, p < 0.05).

**Fig. 3:**
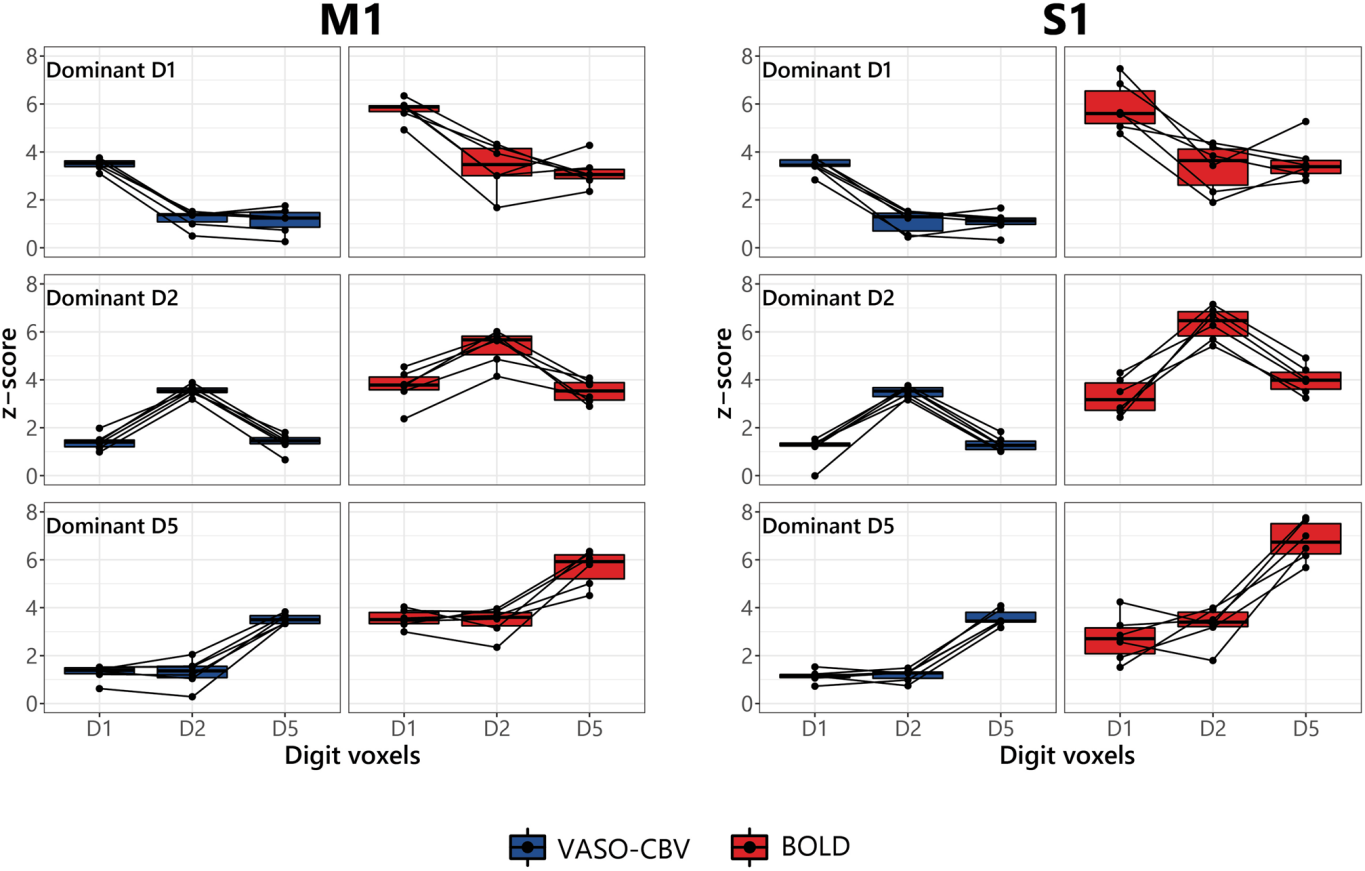
Average z-scores of the dominant finger (columns) in relation to the average z-score of the other voxels (digit voxels). The lines between each digit cluster denote each participant's average z-score. We found similar results for M1 and S1, with higher z-scores for the non-dominant digit voxels in the BOLD data than VASO-CBV data

After assigning the voxels with a winner-take-all strategy, we calculated the average z-scores of the dominant digit for a given digit cluster and the average z-scores of responses for movement of the other digits in the same voxels. Figure 3 illustrates the M1 and S1 results across all six participants. The obtained patterns were consistent across digits and participants, visualized as black dots in each subpanel of Fig. 3. The z-scores for movement of the non-dominant digit were higher in the BOLD data than in the VASO-CBV data for both regions, indicating more overlap and crosstalk in BOLD digit clusters than in VASO-CBV digit clusters, see the average group results in Table 1. These results agree with the DSC and JSC results in Fig. 2 and Supplemental Fig. 3. Consistent with the maps shown in Fig. 1, the z-scores, on the whole, are higher for BOLD.

**Table 1:** Group average (z-scores) of the dominant finger in relation to the digit voxels for M1 and S1

Region	DF	Digit voxels					
		BOLD			VASO-CBV		
		*D1*	*D2*	*D5*	*D1*	*D2*	*D5*
M1	D1	5.76 ± 0.47	3.36 ± 1.01	3.15 ± 0.64	3.48 ± 0.24	1.20 ± 0.39	1.13 ± 0.55
	D2	3.70 ± 0.75	5.37 ± 0.72	3.51 ± 0.48	1.40 ± 0.34	3.55 ± 0.23	1.38 ± 0.39
	D5	3.54 ± 0.38	3.41 ± 0.59	5.66 ± 0.74	1.27 ± 0.34	1.28 ± 0.60	3.53 ± 0.21
S1	D1	5.89 ± 1.05	3.35 ± 1.02	3.60 ± 0.88	3.44 ± 0.34	1.09 ± 0.48	1.07 ± 0.44
	D2	3.29 ± 0.75	6.35 ± 0.69	4.00 ± 0.60	1.12 ± 0.56	3.49 ± 0.24	1.32 ± 0.31
	D5	2.72 ± 0.97	3.28 ± 0.80	6.79 ± 0.83	1.14 ± 0.26	1.18 ± 0.27	3.58 ± 0.35

*DF* Dominant finger

**Fig. 4:**
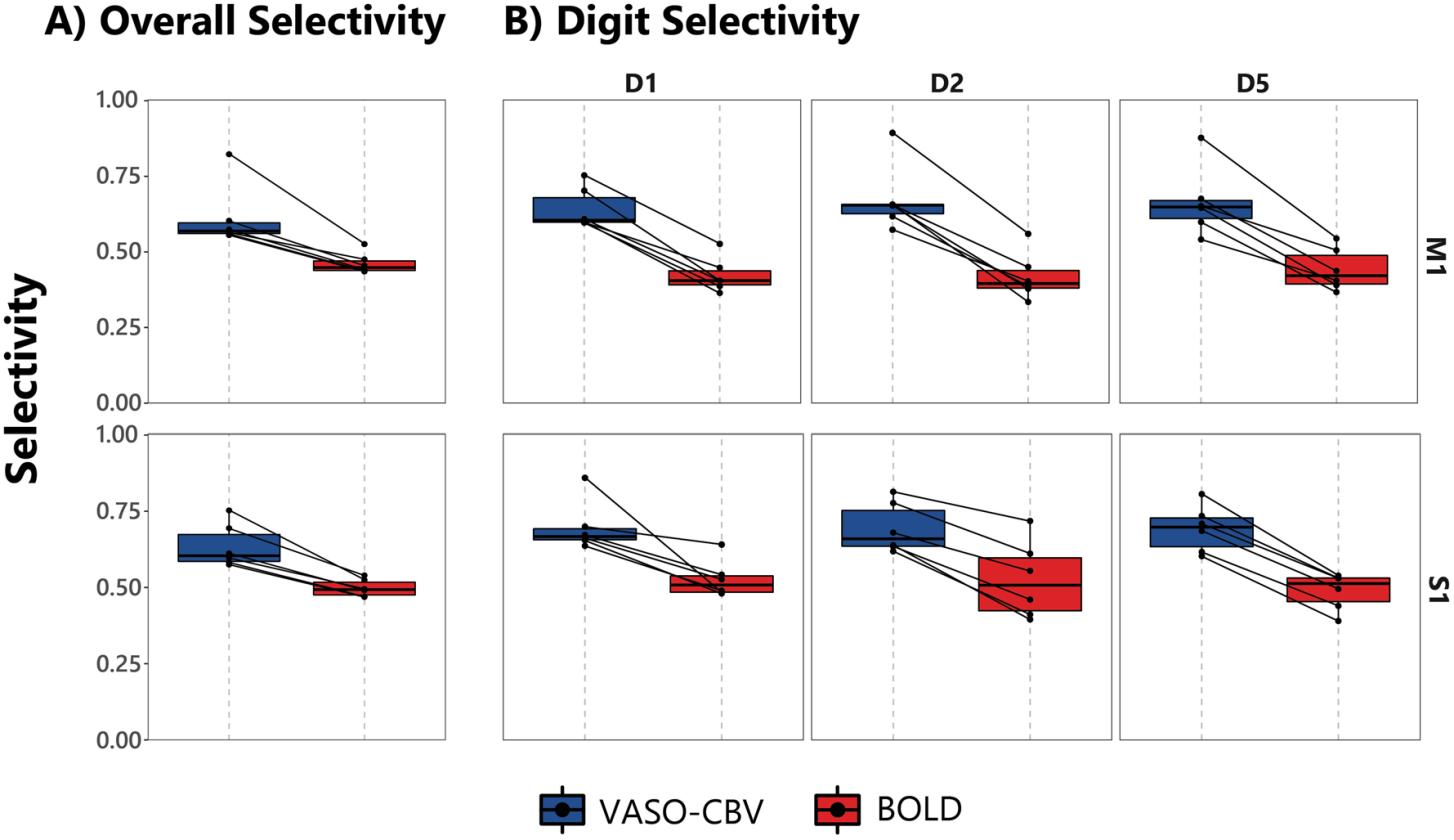
Response selectivity indices for the VASO-CBV and BOLD responses in M1 and S1. **A** Overall selectivity, and **B** Digit selectivity with D1 (thumb), D2 (index), and D5 (little) according to Eqs. 1 and 2 (see Methods). Both selectivity indices describe the magnitude of the fMRI response to the movement of a specific digit relative to the motion of other digits. For the overall selectivity, VASO-CBV showed higher cortical selectivity for all participants (lines denote each participant result) and both M1 and S1. The digit selectivity indices are also higher for VASO-CBV for all participants in both M1 and S1 when compared with BOLD

Figure 4A illustrates the overall selectivity *(OS)* measure for M1 and S1, and Fig. 4B illustrates the digit selectivity (*DS*). For the *OS*, VASO-CBV shows higher selectivity than BOLD for both ROIs (Paired t-test, p < 0.05), with S1 showing higher selectivity indices than M1 for both VASO-CBV and BOLD (average VASO-CBV_M1_ = 0.613 ± 0.104, VASO-CBV_S1_ = 0.636 ± 0.072 and BOLD_M1_ = 0.462 ± 0.035 and BOLD_S1_ = 0.498 ± 0.029). All digit clusters showed similar results for the digit selectivity measure, with VASO-CBV yielding higher selectivity than BOLD (Paired t-test, p < 0.05). Again, S1 yielded higher selectivity than M1 for both VASO-CBV and BOLD, though this difference was not significant (paired t-test, and see Table 2). We also investigated whether different thresholds would produce different results. We observed only small changes for OS, DS, and DICE at the individual level. The observed pattern was consistent across thresholds, with VASO-CBV yielding higher selectivity than BOLD for OS and DS and less overlap than BOLD for the DICE similarity coefficient. See Supplementary Figs. 4, 5 and 6 for more details.

**Table 2:** Average overall (OS) and digit selectivity (DS) for M1 and S1 ROIs (MEAN ± SD)

Region	Method	Digit selectivity			Overall selectivity
		*D1*	*D2*	*D5*	
M1	VASO-CBV	0.642 ± 0.068	0.674 ± 0.111	0.664 ± 0.114	0.613 ± 0.104
	BOLD	0.422 ± 0.058	0.418 ± 0.078	0.441 ± 0.070	0.462 ± 0.035
S1	VASO-CBV	0.696 ± 0.082	0.693 ± 0.082	0.691 ± 0.076	0.636 ± 0.072
	BOLD	0.526 ± 0.061	0.524 ± 0.126	0.487 ± 0.060	0.498 ± 0.029

## Discussion

The present study compares the specificity between VASO-CBV and BOLD cortical activation of individual finger movements in healthy participants employing response selectivity and spatial overlap metrics. We simultaneously measured high-resolution VASO-CBV and BOLD responses using SS-SI VASO sequence with a 3D-EPI readout. Therefore, any observed differences between VASO and BOLD fMRI is unlikely the result of task performance or head movement variations. We assessed the cortical overlap in different ways, (1) by calculating the Dice similarity coefficient (DSC), Jaccard similarity coefficient and assessing crosstalk between digit responses with the averaged z-scores of the dominant finger with respect to the other fingers, and (2) selectivity measurements. Using standard block-designed stimuli, we showed that VASO-CBV yields less overlap between the digit clusters than BOLD for both types of metrics. Moreover, we demonstrate a consistent topographical representation of part of the sensorimotor digit region (thumb-index-little fingers).

We consistently found distinct activation patterns for movement of each of the three digits, organized in an orderly fashion (thumb-index-little) along the central sulcus, reproducing the results reported by Siero et al. (2014), which used the same group of fingers and directional cortical electrophysiological measurements, albeit with a slightly different stimulus design. Moreover, the activation progression pattern was similar to 7 T BOLD fMRI studies employing tactile stimuli (Besle et al. 2014; Kolasinski et al. 2016; Sanchez-Panchuelo et al. 2010; Schellekens et al. 2018; Stringer et al. 2011), 3 T BOLD fMRI (Olman et al. 2012) and a recent 7 T VASO fMRI motor study (Huber et al. 2020). In addition, our results also showed a secondary representation of the index and thumb, positioned superior to the little finger representations (seen for participants P02-P05), as previously found by (Huber et al. 2020). The VASO-CBV responses are smaller in amplitude and cluster size than the BOLD responses, as also reported previously in different VASO-CBV—BOLD comparisons (Huber et al. 2020, 2017; Oliveira et al. 2022, 2021).

For the DSC results, the BOLD data yielded higher DSC values than VASO-CBV and hence higher overlap between the activation clusters. The results for the Jaccard similarity coefficient were nearly identical (see Supplementary Fig. 3). A complementary measurement was performed with the averaged z-scores of the dominant digit relative to the average z-scores of the responses for the other digits (Fig. 2). The averaged z-scores behaved similarly to the overlap metrics (DSC and JSC). Moreover, our results were consistent across participants. VASO-CBV yielded higher selectivity than BOLD for the overall and the individual digit selectivity (Fig. 4). These results were also consistent across participants. We did not observe significant differences between M1 and S1 in the DSC, JSC, or crosstalk measures except in (D2-D5, for DSC). Taken together, the observed higher response selectivity for the VASO-CBV signal corroborates the hypothesized higher vascular specificity of VASO-CBV fMRI compared to the BOLD signal.

The digit overlap between different digit representations has different sources, i.e., neural, vascular, and methodology-related overlap. Because we acquired VASO-CBV and BOLD signal within the same functional run, the neural and methodological contributions are not expected to differ in VASO-CBV compared to BOLD. The higher specificity in VASO-CBV is likely due to the lower vascular contribution in the point-spread function. Thus, we speculate that the selectivity quantification using VASO-CBV could be a tool to investigate pathological conditions related to sensory and motor function and may perhaps be used to investigate brain plasticity or aging and development.

Regarding the selectivity metrics used, we opted for multiple selectivity metrics rather than a single metric because there are pros and cons to applying these metrics. As our results show, *OS* and *DS* metrics demonstrate a higher spatial selectivity for the VASO-CBV responses. However, by using the overall selectivity, lower or negative responses (z-scores) in a single-digit voxel can lead to divisions by zero and outliers values, potentially shifting the ROI selectivity value. We did not observe a significant number of outliers here (less than 1%). On the other hand, digit selectivity is better controlled, and this metric captures the selectivity for each digit. For these reasons, we favor *digit selectivity* rather than *overall selectivity*. Nevertheless, both metrics showed the same pattern and were consistent across participants. Another common approach is calculating the sum of the absolute value of the responses using Eq. 1. This way, the outliers can also be avoided; however, it can also lead to overestimating or underestimating the selectivity values (see *OS*_*2*_ in Supplementary Fig. 1).

## Conclusion

Here, we simultaneously recorded submillimeter VASO-CBV and BOLD signals to investigate cortical activation in the primary somatosensory and motor areas during individual finger movement. We used similarity and response selectivity metrics to compare VASO-CBV and BOLD specificity. BOLD showed a higher overlap and lower selectivity than VASO-CBV. These results were consistent across metrics and participants. Together, they suggest that the higher vascular specificity of VASO-CBV results in higher response selectivity or less vascular overlap than BOLD, confirming the higher spatial specificity of VASO-CBV compared to BOLD.

## Supplementary Information

Below is the link to the electronic supplementary material.

Supplementary file1 (PDF 1432 KB)

## Data Availability

Raw MRI data will be available upon request to the authors. MRI data is considered personal data pursuant to General data protection regulation (GDPR) and can only be shared based on and subject to the Royal Netherlands Academy of Arts and Sciences (KNAW) policies. Considering the requirements imposed by law and the sensitive nature of personal data, any requests will be addressed on a case-by-case basis, subject to a data usage agreement.

## Abbreviations

fMRI: Functional magnetic resonance imaging
UHF: Ultra-high field
tSNR: Temporal signal-to-noise ratio
VASO: Vascular space occupancy
BOLD: Blood oxygenation-level dependent
EPI: Echo planar imaging
GRE: Gradient-echo
ASL: Arterial spin labeling
CBV: Cerebral blood volume
CBF: Cerebral blood flow
PVE: Partial volume effect
PCA: Principal component analysis
