# Supplementary material for: Improved Selectivity in 7 T Digit Mapping Using VASO-CBV
Source: Brain Topogr. 2022 Dec 14;36(1):23–31. doi: 10.1007/s10548-022-00932-x (PMC9834127; doi:10.1007/s10548-022-00932-x)
Supplement: Supplementary file 1 — Supplementary file1 (PDF 1432 KB) [file 10548_2022_932_MOESM1_ESM.pdf]

Supplementary material

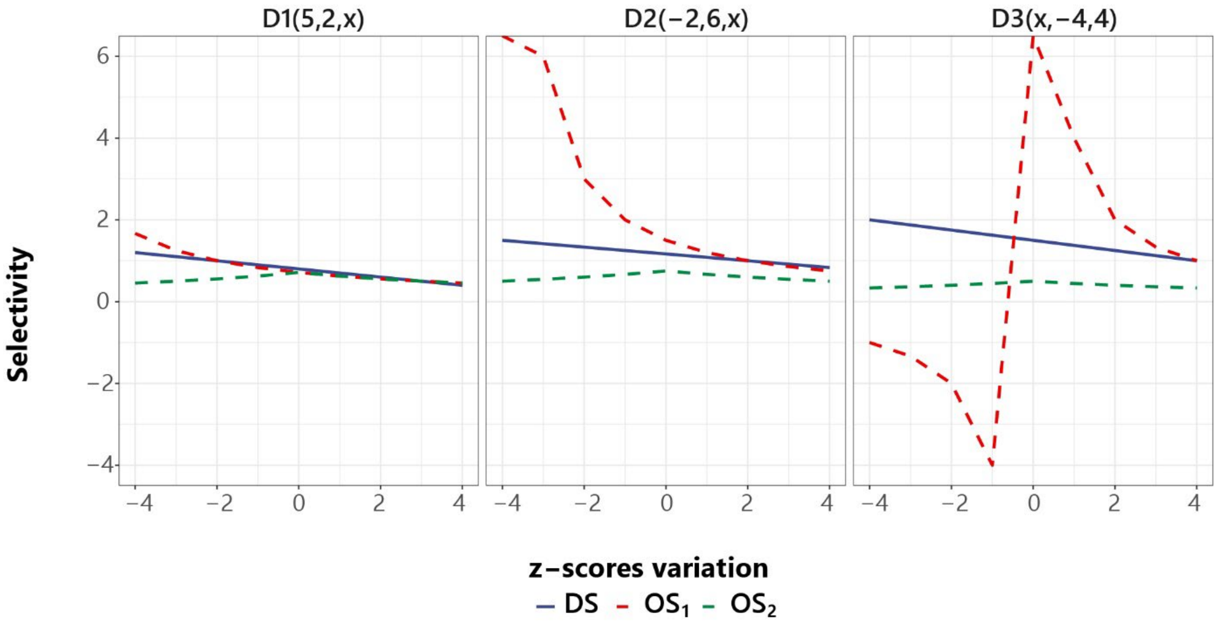

Supplementary Figure 1: Selectivity response using two variants of the overall selectivity (OS_1 and OS_2) in addition to the digit selectivity (DS). We computed each metric using the z-scores of the three fingers, D1, D2, and D5. Each column represents the result in a specific dominant digit. The number in parenthesis represents the z-scores, and x represents the digit z-score that we vary from -4 to 4. Each curve highlights the advantages and disadvantages of each selectivity method. For example, OS_1 is attractive because of its straightforward computation; it does not require a winner-take-all step. However, lower z-scores or negative values can lead to divisions by zero or negative selectivity responses, acting as outliers as shown for D2 and D3). In OS_2, the sum of the digits uses the absolute value of each digit. Thus the OS_2 can avoid the outliers because the division by zero is not possible anymore. However, it can also lead to underestimating the selectivity values. The *DS* metric shows a more linear behavior, which we believe is preferred. The mean difference between the target digit and the others avoids division by zeros, and the *DS* can capture the selectivity of each digit rather than just one overall measurement.

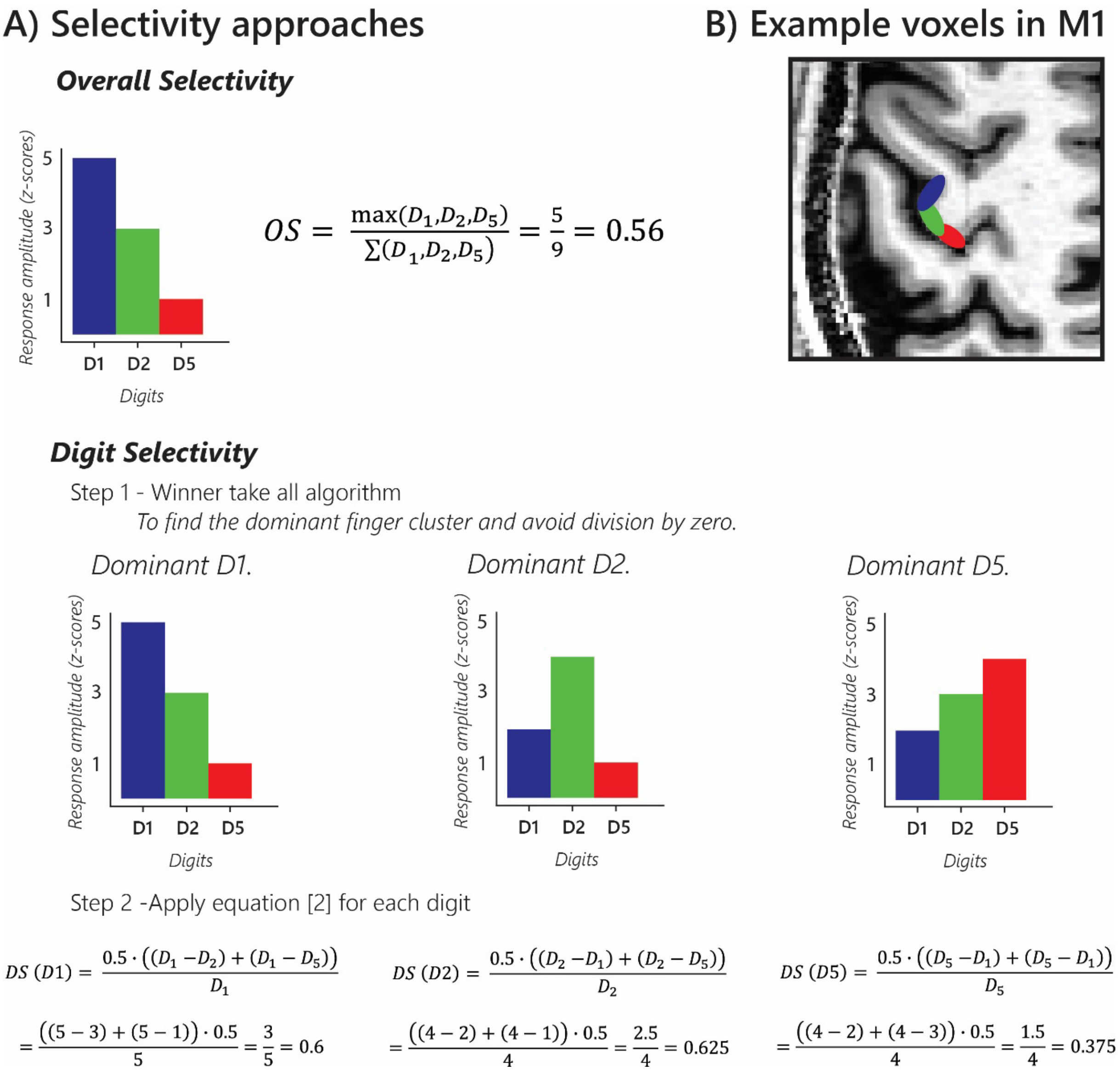

Supplementary Figure 2: Graphical explanation of the selectivity metrics used in the present study. Panel A) shows both Selectivity approaches in detail. Panel B) shows example voxels in M1. The general approach is called 'overall selectivity' (*OS*). For the *OS* approach, we divide the maximum response amplitude (z-scores) between all three digits by the sum of the responses to all three-digit movements per voxel. The second approach is called 'digit selectivity' (*DS*). Voxels were assigned to a specific digit for the DS approach using a winner-takes-all approach (first step). The second step consisted of the calculation of the *DS* using the Equation 2. This method takes the mean difference between the responses of the dominant digit in relationship to the other two digits divided by the response of the dominant finger.

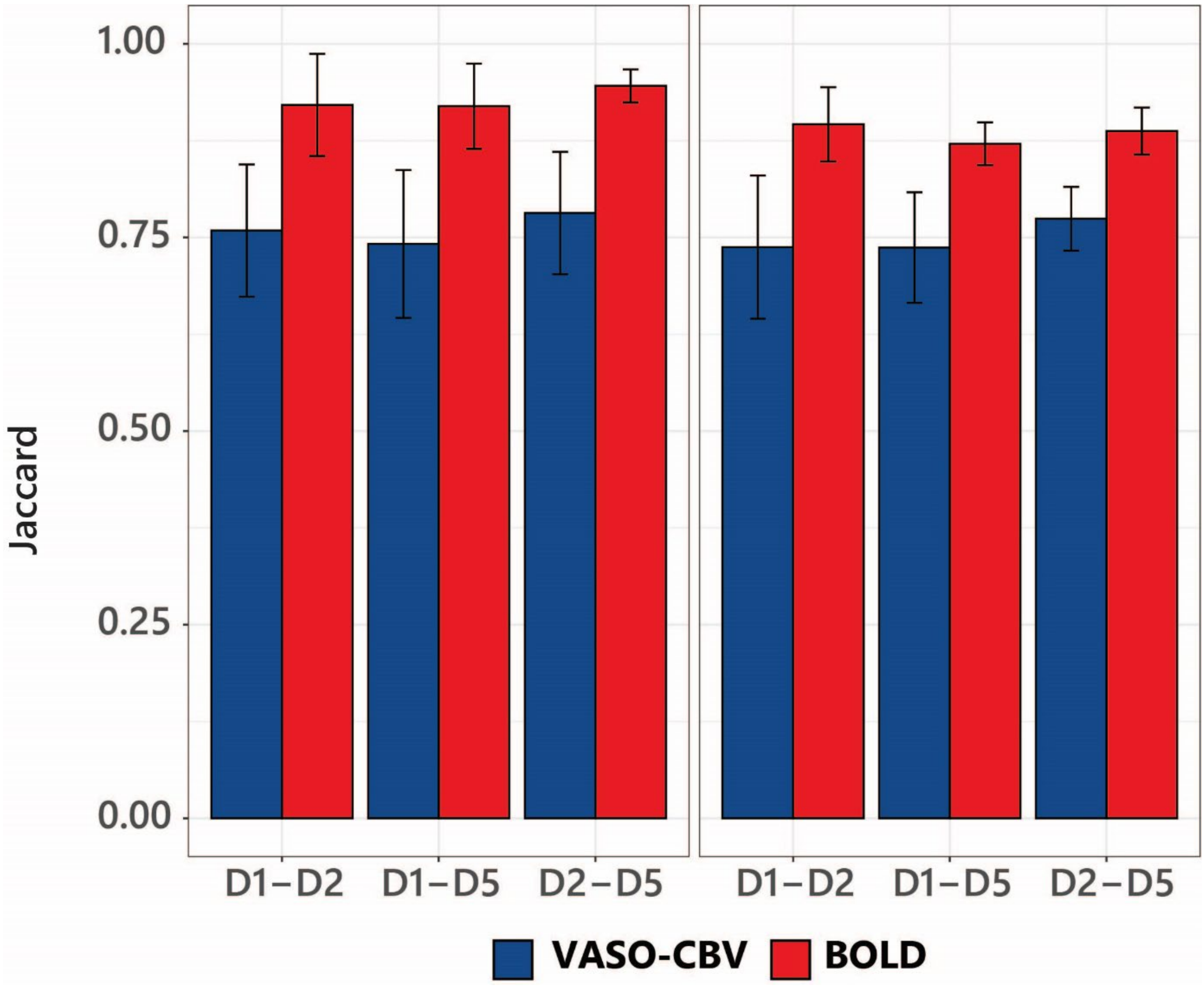

Supplementary Figure 3: Jaccard Similarity Coefficient (JSC) for each pair-digit in S1 and M1. As seen in Figure 2 with the DICE coefficient, the BOLD responses yielded higher scores than VASO-CBV (Paired t-test, $p<0.05$) in both regions. A higher similarity score represents a higher overlap between each digit pair. The error bar is the standard deviation.

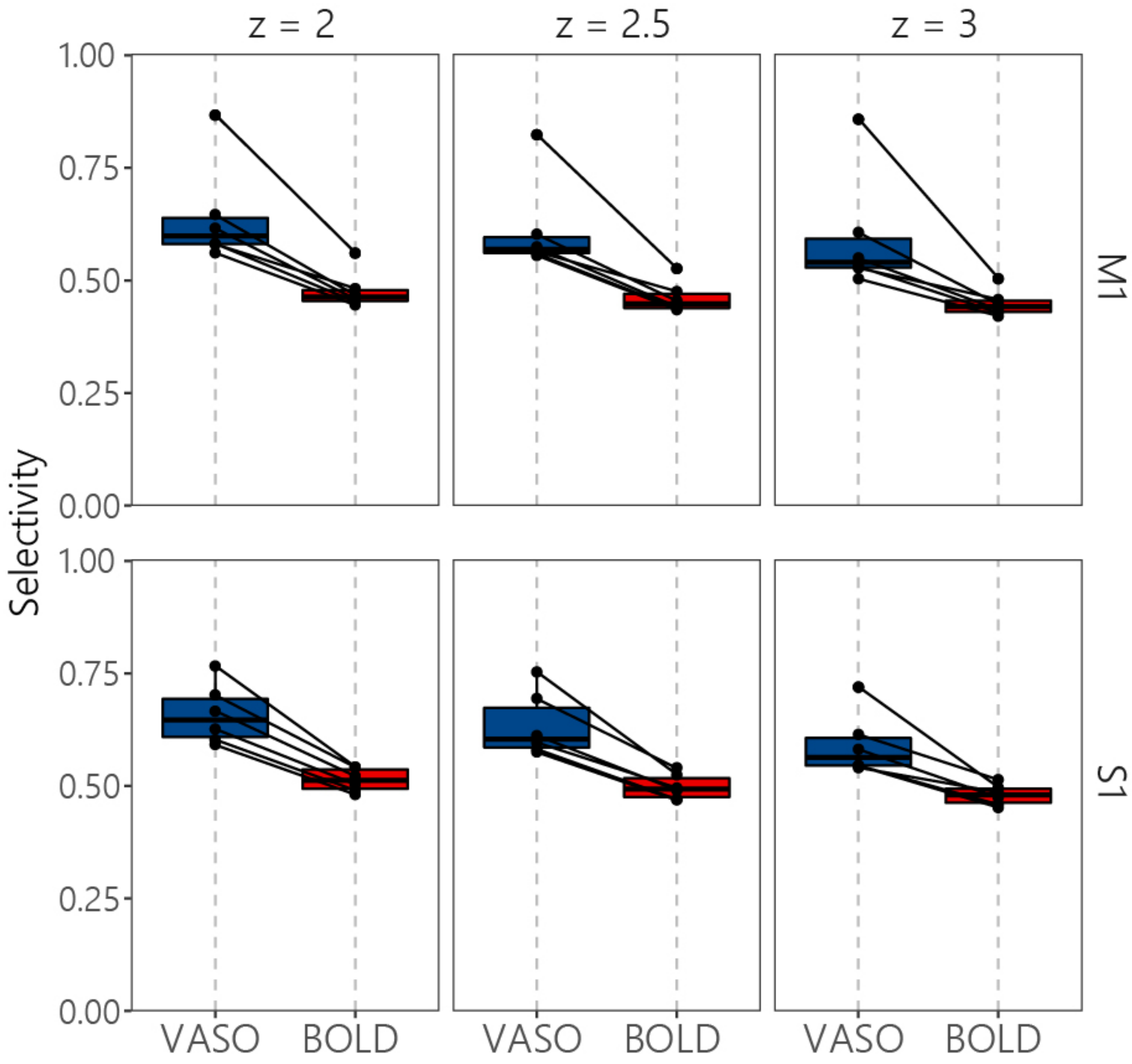

Supplementary Figure 4: Overall Selectivity comparison between VASO-CBV and BOLD for different z-score thresholds. z=2, z=2.5 and z=3. Increasing the threshold does not change the relationship between VASO-CBV and BOLD. VASO-CBV consistently yields higher selectivity than BOLD across participants, regions, and thresholds.

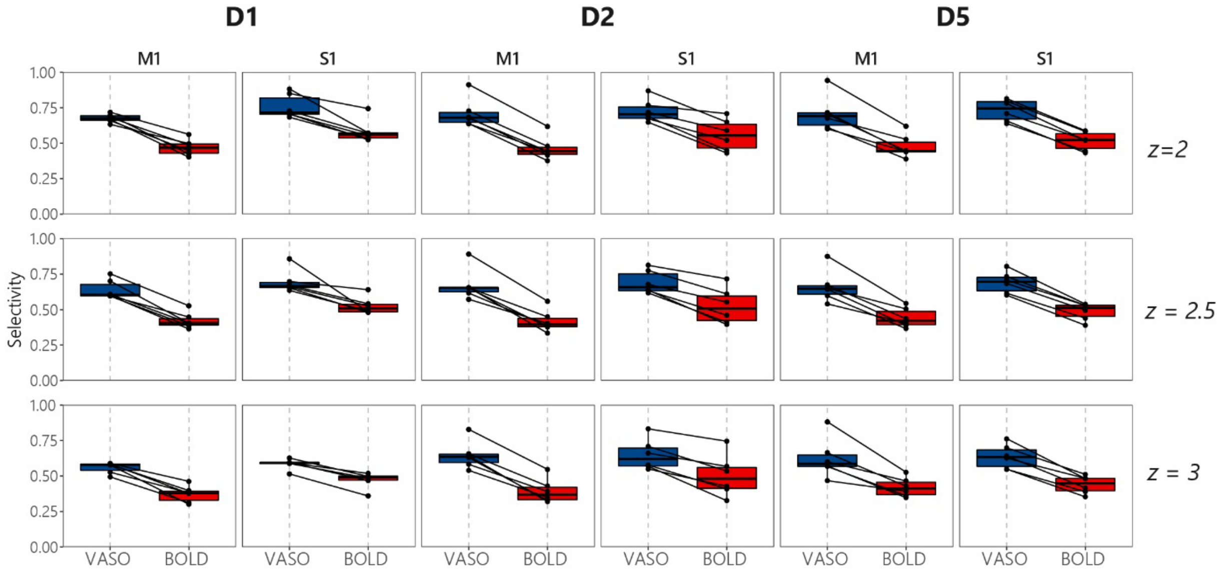

Supplementary Figure 5: Digit Selectivity comparison between VASO-CBV and BOLD for different z-score thresholds. z=2, z=2.5 and z=3. Increasing the threshold does not change the relationship between VASO-CBV and BOLD. VASO-CBV consistently yields higher selectivity than BOLD across participants, regions, and thresholds.

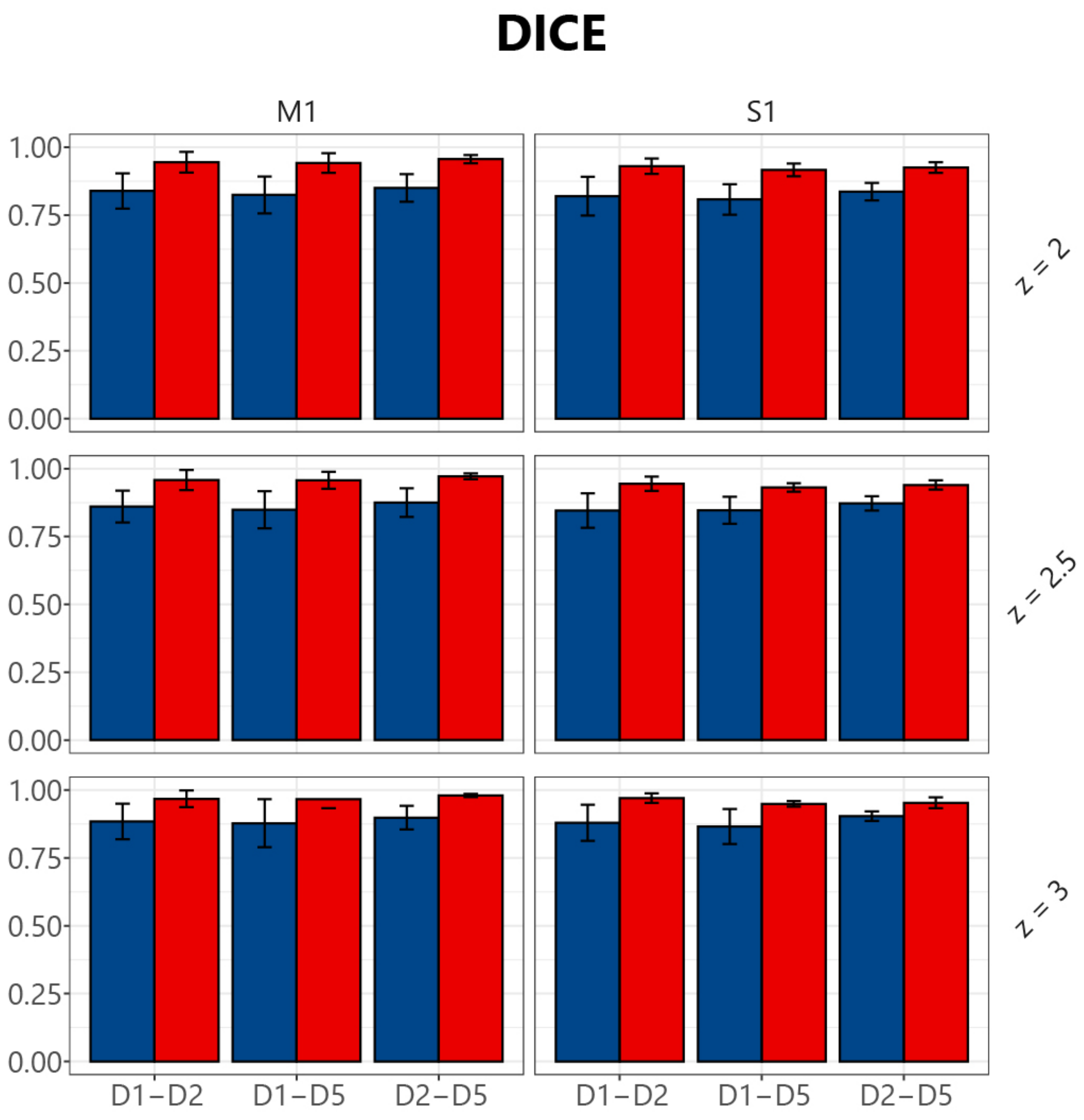

Supplementary Figure 6: DICE Similarity coefficient comparison between VASO-CBV and BOLD for different z-score thresholds. z=2, z=2.5 and z=3. Increasing the threshold does not change the relationship between VASO-CBV and BOLD. BOLD consistently yields higher overlap (DICE) than BOLD across participants, regions, and thresholds.
